# Primary Candida glabrata Infection of a Pancreatic Pseudocyst

**DOI:** 10.7759/cureus.82248

**Published:** 2025-04-14

**Authors:** Helen Bolanaki, George Pappas Gogos, Panagoula Oikonomou, Ioannis Tzimagiorgis, Anastasios J Karayiannakis

**Affiliations:** 1 Second Department of Surgery, University Hospital of Alexandroupolis, Alexandroupolis, GRC; 2 Second Department of Surgery, University Hospital of Alexandroupolis/Democritus University of Thrace, Alexandroupolis, GRC

**Keywords:** antifungals, candida glabrata, drainage, echinocandins, pancreatic pseudocyst

## Abstract

This study describes a case of an 80-year-old patient admitted to our hospital with mid-epigastric pain, nausea, vomiting, and persistent fever for the last two days, after an episode of acute gallstone pancreatitis six weeks earlier. On admission, the patient was clinically stable with leukocytosis, high serum amylase levels, and high CRP levels. An abdominal CT scan showed a well-circumscribed, thick-walled fluid collection with air-fluid levels suggestive of an infected pancreatic pseudocyst. The patient underwent exploratory laparotomy in which fluid aspiration, removal of necrotic tissue, placement of drainage tube, and cholecystectomy were performed. Cultures from the pseudocyst revealed *Candida glabrata*. Antibiotics were discontinued, and intravenous antifungal therapy with anidulafungin was initiated at a loading dose of 200 mg, followed by a maintenance dose of 100 mg daily. The patient's condition improved shortly thereafter, with resolution of fever and normalization of laboratory tests. After three weeks of antifungal therapy, with no positive cultures from the drainage tube and no pathological findings on repeat CT, the patient was discharged from the hospital. He was doing well at six- and 12-month follow-ups.

## Introduction

Pseudocyst formation after an episode of severe acute necrotizing pancreatitis (AP) is not uncommon. Approximately half of pancreatic pseudocysts will become symptomatic or complicated by infection, rupture, hemorrhage, vascular thrombosis, or obstruction of adjacent structures. The most commonly isolated microorganisms from an infected pseudocyst are gastrointestinal Gram-negative bacteria, such as *Escherichia coli*, species of Klebsiella and Enterococci, and *Streptococcus faecalis,* suggesting their translocation from the adjacent large bowel [1-3].

Fungal infections, in the forms of infected necrosis, abscess, or infected pseudocyst, once considered very rare, are increasingly being reported and associated with increased morbidity and mortality. Fungal infections are most frequently caused by *Candida albicans*, usually as part of a polymicrobial infection, followed by *Candida tropicalis* and *Candida parapsilosis,* whereas there have been very few cases of pancreatic pseudocysts infected by *Candida glabrata* [4,5]. Herein, we describe a patient in whom *Candida glabrata* was the only pathogen isolated from an infected pancreatic pseudocyst formed after an episode of acute necrotizing pancreatitis.

## Case presentation

An 80-year-old man was admitted to our hospital with mid-epigastric pain, nausea, vomiting, and persistent fever over the last two days. Six weeks before his admission, he was diagnosed with acute gallstone pancreatitis and was treated conservatively in a community hospital. An intravenous contrast-enhanced abdominal CT scan performed at that time revealed diffusely enlarged pancreas, peripancreatic inflammatory changes, and a single, lesser-sac fluid collection, and the pancreatitis was classified as grade D according to the Balthazar classification criteria. According to his notes, the patient was treated with intravenous fluids and bowel rest. Intravenous imipenem/cilastatin sodium and amikacin sulphate were given for 10 days when his clinical condition was improved, the laboratory tests were normal, and the pancreatic and peripancreatic inflammatory changes and the fluid collection on a repeat CT scan were resolved. He was discharged after 12 days of hospitalization with a recommendation for planned cholecystectomy after eight weeks.

On admission, the patient appeared ill but was clinically stable. His blood pressure was 110/70 mmHg with a pulse rate of 100/min. His body temperature was 38.5°C. Abdominal distension, diffuse tenderness in the upper abdomen, and a slightly tender palpable epigastric mass of elastic consistency were found on physical examination.

Laboratory tests revealed mild anemia (decreased red cell count and hematocrit) as well as low levels of hemoglobin. On the other hand, the patient expressed marked leukocytosis with increased levels of neutrophils, and elevated serum concentration of C-reactive protein (CRP). Serum amylase level was elevated, but urinary amylase was normal (Table 1). Liver function tests revealed high levels of aspartate aminotransferase (AST), alanine aminotransferase (ALT), γ-glutamyl transpeptidase (γGT), alkaline phosphatase (ALP), total bilirubin (TBL), and direct bilirubin (DBL). Total serum protein (TPR) levels were within normal range, but albumin (ALB) levels were decreased. Platelet count, prothrombin time, serum glucose and electrolyte levels, and renal function tests were all normal (Table 1).

**Table 1:** Quantitative laboratory investigations of the patient. Ht: hematocrit; Hb: hemoglobin; PLT: platelets; AMY: amylase; AST: aspartate aminotransferase; ALT: alanine aminotransferase; γGT: γ-glutamyl transpeptidase; ALP: alkaline phosphatase; TBL: total bilirubin; DBL: direct bilirubin; TPR: total serum protein; ALB: albumin; GLU: glucose; Cre: creatinine

Laboratory tests	Value	Reference range
RBC	5.23×10^6^/mm^3^	4.5-6.5×10^6^/mm^3^
Ht	34.6%	40-54%
Hb	11.4 g/dL	13.5-17.5 g/dL
WBC	17,260/mm^3^	3,500-10,800/mm^3^
Neutrophils	89.7%	40-75%
PLT	258,000/mm^3^	150,000-400,000/mm^3^
CRP	16.25 mg/dL	<0.5 mg/dL
AMY (serum)	1,283 U/L	28-100 U/L
AMY (urine)	238 U/L	42-321 U/L
AST	64 U/L	<35 U/L
ALT	52 U/L	<45 U/L
γGT	65 U/L	9-55 U/L
ALP	152 U/L	152 U/L
TBL	1.3 mg/dL	0.3-1.2 mg/dL
DBL	0.4 mg/dL	<0.2 mg/dL
TPR	6.5 g/dL	6-8.3 g/dL
ALB	3.1 g/dL	3.5-5.2 g/dL
GLU	93 mg/dL	70-100 mg/dL
Na^+^	138 mmol/L	135-145 mmol/L
K^+^	4.2 mmol/L	3.6-5.2 mmol/L
Urea	14 mg/dL	7-20 mg/dL
Cre	0.8 mg/dL	0.7-1.3 mg/dL

**Figure 1:**
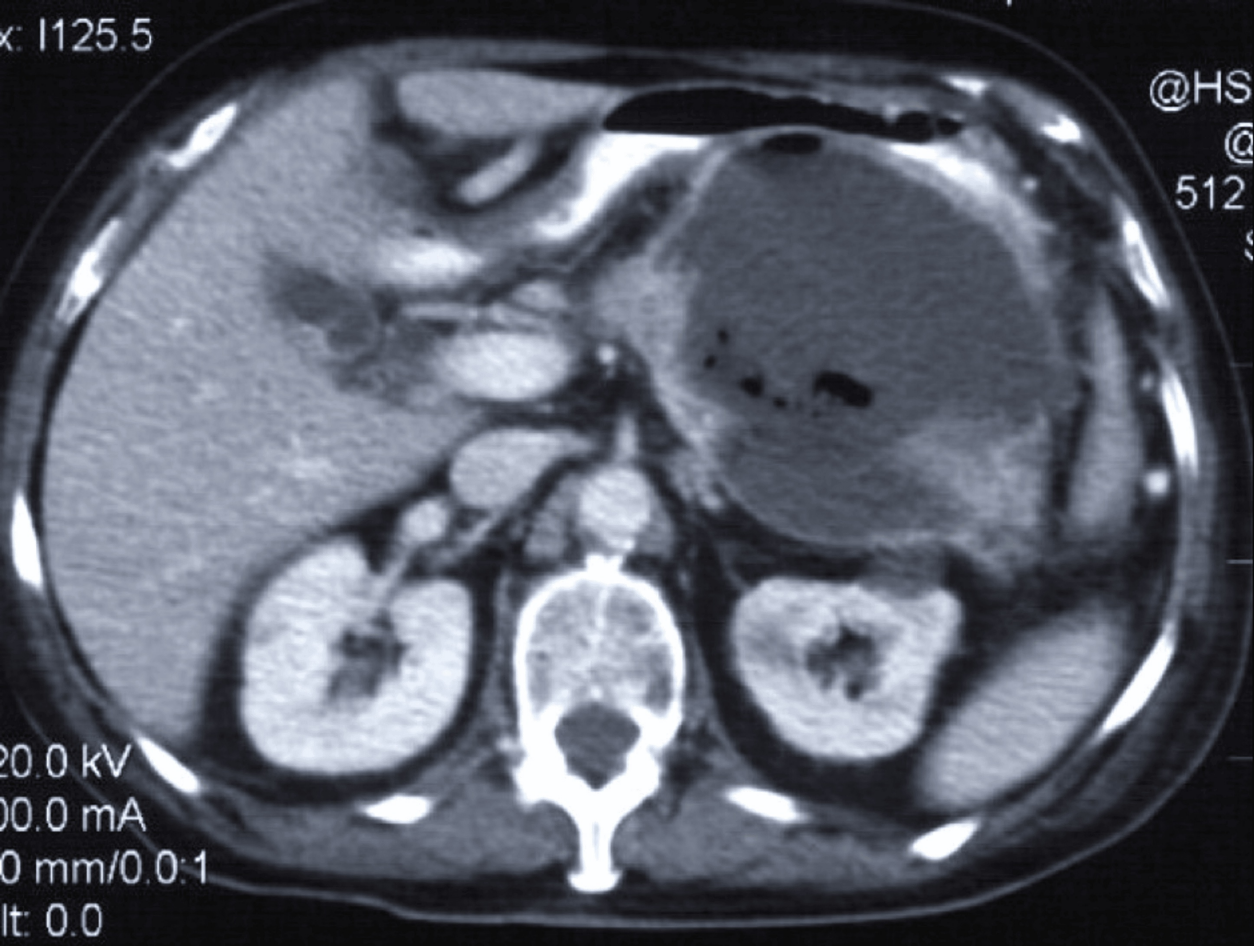
Enhanced computed tomography showing a well-circumscribed, thick-walled pancreatic pseudocyst compressing the gastric wall. Cyst wall enhancement and gas within the cyst suggest an infected pancreatic pseudocyst.

Abdominal ultrasonography confirmed cholelithiasis without dilatation of the intra- or extrahepatic biliary tree or choledocholithiasis. A thick-walled cyst was also found in the body of the pancreas. Computed tomography (CT) showed a well-circumscribed, thick-walled fluid collection with gas bubbles and air-fluid levels suggestive of an infected pancreatic pseudocyst (Figure 1).

The patient was empirically initiated on amikacin, ampicillin/sulbactam, and metronidazole. Percutaneous drainage under CT guidance was considered, but a safe access route was deemed unlikely. The patient underwent an exploratory laparotomy. After division of the gastrocolic ligament, the pseudocyst was accessed, opened and a fluid sample was sent for biochemistry and culture. The fluid was aspirated, sequestered necrotic tissues were removed, and a large-bore catheter was left in the pseudocyst for external drainage. Cholecystectomy was also performed. Cultures revealed *Candida glabrata* but no bacteria. Repeated blood and urine cultures were negative for aerobes, anaerobes, and fungi. Administration of antibiotics was discontinued and intravenous antifungal therapy with anidulafungin was initiated with a loading dose of 200 mg, followed by daily 100-mg maintenance. By the third day of treatment the patient showed a marked improvement with resolution of the fever. His laboratory tests normalized 10 days later. After three weeks of antifungal therapy, cultures of the draining fluid revealed *Staphylococcus epidermidis* but no pathogenic bacteria or fungi, the amylase level was 68 U/L, and there were no pathological findings on a repeat CT scan. The drainage tube was removed and the patient was discharged from the hospital. He was doing well at the time of follow-ups at six and 12 months.

## Discussion

Pancreatic pseudocysts represent organized collections after an episode of acute necrotizing pancreatitis when the peripancreatic fluid collections do not resolve and a fibrotic capsule is formed around them by a reactive inflammatory tissue. They usually develop four to six weeks after the initial attack and should be differentiated from the fluid collections and walled-off necroses, which occur early after an episode of acute pancreatitis [1,2].

A pancreatic pseudocyst may become symptomatic or complicated if not spontaneously resolved. Infection of a pseudocyst is a frequent and serious complication. Most often, multiple microbial species are isolated from an infected pseudocyst as opposed to infected pancreatic necrosis, where monomicrobial infections are usually present. Gram-negative bacteria like *Escherichia coli*, *Klebsiella spp.*, *Enterococcus spp.,* and *Enterobacter spp.* are most commonly isolated, suggesting their translocation from the adjacent large bowel, although hematogenous or lymphatic spread from other sites cannot be excluded. Fungal infections are most frequently caused by *Candida albicans*, followed by *Candida tropicalis *and *Candida parapsilosis,* whereas there have been very few cases of pancreatic pseudocysts infected by *Candida glabrata* [3-5].

*Candida glabrata*, also known as Torulopsis glabrata, is a normal commensal in the gastrointestinal and genitourinary tract and in the skin. It is a non-dimorphic, haploid yeast, which does not form hyphae but reproduces by budding and exists as small blastoconidia. Infections of the necrotic pancreatic tissue or of a pancreatic pseudocyst with *Candida glabrata *have been previously reported [5-7]. The exact mechanism of fungal infection of a pancreatic pseudocyst is unclear; however, lymphatic spread or translocation from the colon is assumed, whereas hematogenous spread is less likely [8]. Several factors, such as previous interventional procedures like debridement of necrotic tissue or drainage of a fluid collection or pseudocyst, are potential risk factors. Prolonged hospitalization and prophylactic administration of broad-spectrum antibiotics in patients with acute necrotizing pancreatitis are other major risk factors for colonization of the necrotic tissue with fungi, either as the only pathogen or in combination with other bacteria, with up to 40% incidence of fungal contamination in some series [5,8]. Our patient had both of these risk factors. During his previous 12-day hospitalization, he was treated with imipenem/cilastatin sodium and amikacin sulphate for 10 days.

Fungal infection of pancreatic necrosis associates with increased mortality when compared to bacterial only infections [6]. Therefore, prompt diagnosis is important. In a patient with a previous attack of acute necrotizing pancreatitis, when clinical signs and laboratory findings such as fever and leukocytosis suggest infection, a contrast-enhanced CT is useful in making the correct diagnosis. Pseudocyst wall enhancement and the presence of gas within the cyst are characteristic features of an infected pseudocyst. Drainage of the pseudocyst, either surgically or percutaneously, is essential and is decided on an individual basis. Cultures of the material from the pseudocyst taken either during open surgery or by CT-guided needle aspiration should include testing for both bacterial pathogens and fungi. As the number of fungal infections is increasingly reported, early and rapid detection of *Candida glabrata* is necessary for prompt treatment [5,7,9].

Blood cultures for Candida species are less sensitive as the yeast is rapidly cleared from the circulation. Therefore, yeast isolation in the pseudocyst material is essential for the diagnosis of fungal infection, especially when it is the only organism isolated. When both fungi and bacteria are isolated, then fungal infection should be considered if there are clinical signs and laboratory findings of systemic infection [5,7-9].

Both amphotericin B and fluconazole may be used for the medical treatment of a pancreatic pseudocyst infected with *Candida glabrata*. Amphotericin B incorporates into the cytoplasmic membrane of the yeast and increases its permeability thus resulting in fungal death. It is very effective in cases of systemic infections caused by *Candida glabrata*. Nephrotoxicity is a significant side effect and should be considered in patients with impaired renal function. Fluconazole is also successful against Candida species infections. Fluconazole interacts with a number of membrane constituents, causing an impairment of function and cessation of growth. Side effects are mild and include mainly nausea, abdominal pain, and diarrhea [5].

However, *Candida glabrata* resistance to fluconazole is not unusual. *Candida glabrata* has been increasingly reported as an important nosocomial pathogen of special interest because of its inherent resistance to antifungal treatment with azoles [10]. In our patient, monotherapy with anidulafungin was given after isolation of *Candida glabrata* in the pancreatic pseudocyst and was very effective with rapid clinical improvement of the patient. This antifungal medicine belongs to the group of echinocandins and acts by interfering with the production of glucan, a component of the fungal cell wall, which is necessary for the fungus's survival. It is used for the treatment of invasive candidiasis in adults who are not neutropenic and is considered safe with minor effects on renal and hepatic function. At least two weeks of treatment is recommended after the detection of Candida species. Thereafter, depending on the patient's response, treatment may be continued until complete symptom improvement and no further isolation of the fungus. *Candida glabrata* resistance to antifungal treatment with azoles has been increasingly reported, making treatment with anidulafungin a useful alternative. Although anidulafungin has been shown to be more effective than fluconazole, there have been reports of *Candida glabrata* strains resistant to echinocandins along with multidrug resistance to several antifungal agents [11,12].

## Conclusions

This study aimed to highlight the increasing incidence of fungal infections in patients with severe pancreatitis complicated by pseudocyst formation. It should also be noted that the shift towards non-albicans Candida species, such as *Candida glabrata*, along with the development of resistance to several antifungal agents, represents an emerging concern for healthcare worldwide.

In patients with infected pancreatic pseudocysts after an episode of acute necrotizing pancreatitis, fungal infections should be suspected and promptly investigated. Early and rapid detection of *Candida glabrata* is essential for timely treatment with appropriate antifungal agents, considering the inherent resistance to some antifungals.
